# Supplementary material for: Neoadjuvant Chemotherapy of Triple-Negative Breast Cancer: Evaluation of Early Clinical Response, Pathological Complete Response Rates, and Addition of Platinum Salts Benefit Based on Real-World Evidence
Source: Cancers (Basel). 2021 Mar 30;13(7):1586. doi: 10.3390/cancers13071586 (PMC8036281; doi:10.3390/cancers13071586)
Supplement: Supplementary file 1 [file cancers-13-01586-s001.pdf]

cancers

Supplementary Materials

Neoadjuvant Chemotherapy of Triple-Negative Breast Cancer: Evaluation of Early Clinical Response, Pathological Complete Response Rates, and Addition of Platinum Salts Benefit Based on Real-World Evidence

Milos Holanek, Iveta Selingerova, Ondrej Bilek, Tomas Kazda, Pavel Fabian, Lenka Foretova, Maria Zvarikova, Radka Obermannova, Ivana Kolouskova, Oldrich Coufal, Katarina Petrakova, Marek Svoboda and Alexandr Poprach

BRCA germline mutation testing categorization

Out of a total of 237 patients, 72 (30%) patients were *BRCA1/2* mutated, 109 (46%) patients were *BRCA1/2* unmutated, and in 56 (24%) patients, the genetic testing was not performed. The age distribution according to *BRCA* testing is shown in Figure S1A. The median age was 37 years in the *BRCA1/2* mutated patients, 44 years in the were *BRCA1/2* unmutated patients and 63 years for untested patients. The untested patients were not indicated to genetic testing following the guidelines for high-risk patients, especially family cancer history was not described. According to a published study[1], the expected prevalence of *BRCA1/2* carriers in women with TNBC diagnosed at age 50 or above is less than 5%. Consequently, the untested patients were considered presumptive noncarriers and analyzed together with *BRCA1/2* noncarriers. Similarly, in the case of unknown *BRCA* status in daily clinical practice, these patients are considered *BRCA1/2* negative.

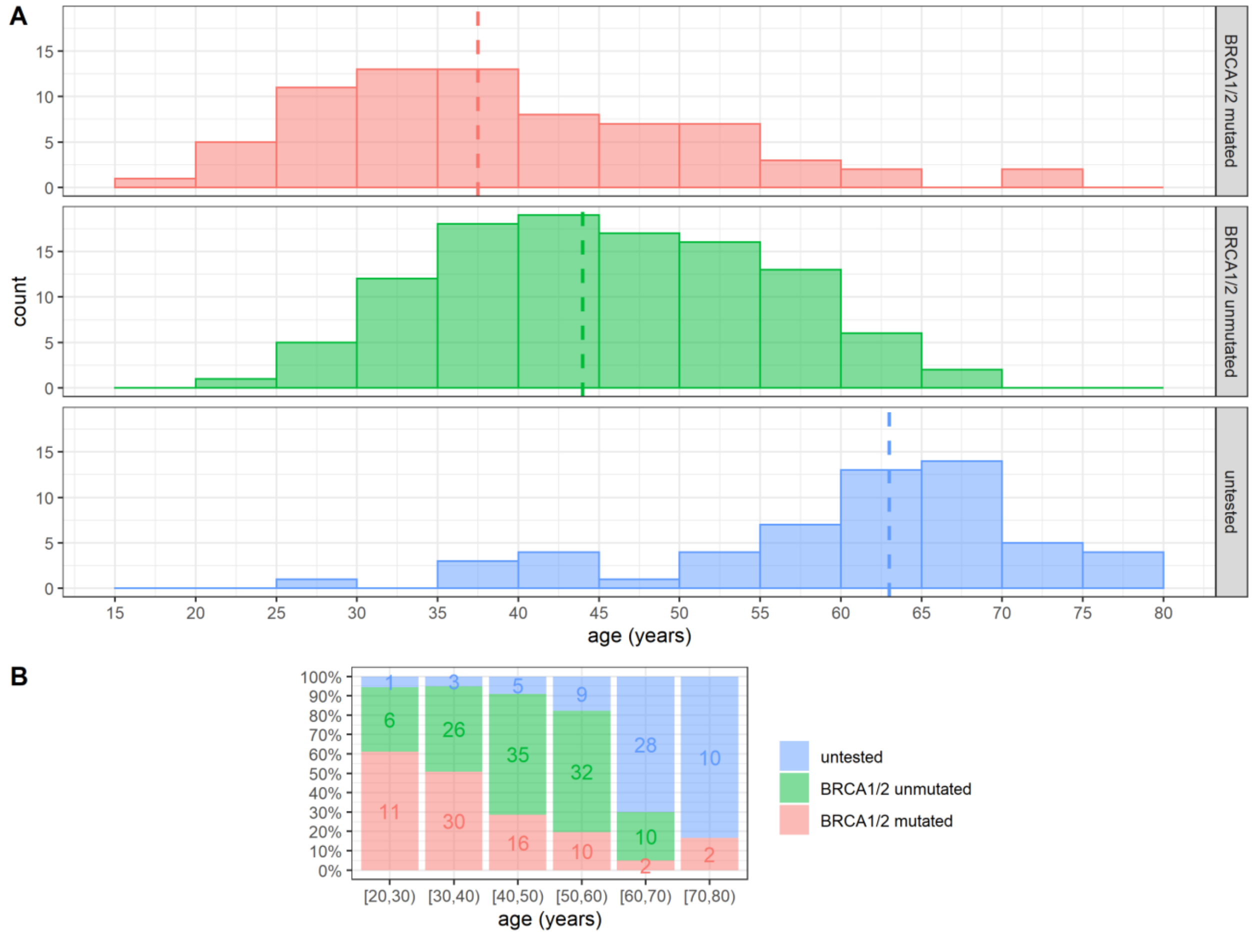

Figure S1. (**A**) Age distribution according to *BRCA* testing. (**B**) The proportion of *BRCA1/2* mutation in age decade.

1. Engel, C.; Rhiem, K.; Hahnen, E.; Loibl, S.; Weber, K.E.; Seiler, S.; Zachariae, S.; Hauke, J.; Wappenschmidt, B.; Waha, A.; et al. Prevalence of Pathogenic BRCA1/2 Germline Mutations among 802 Women with Unilateral Triple-Negative Breast Cancer without Family Cancer History. BMC Cancer 2018, 18, doi:10.1186/s12885-018-4029-y.

Table S1. ECR-adjusted univariable analysis of pCR predictors according to NACT type. NACT, neoadjuvant chemotherapy; BMI, Body mass index

	NACT	N	OR	95% CI	*p*	*p* for interaction
**NAT** platinum / nonplatinum		68 / 169	3.14	[1.57;6.58]	**<0.001**	
**Early clinical response**						0.896
yes / no	platinum	43 / 25	21.33	[6.01;103.5]	**<0.001**	
	nonplatinum	126 / 43	18.64	[5.41;117.4]	**<0.001**	
**Age (10-years)**						0.200
	platinum	68	1.30	[0.74;2.36]	0.362	
	nonplatinum	169	0.85	[0.65;1.11]	0.244	
**Age (years)**						**0.016**
≥ 45 / < 45	platinum	24 / 44	4.53	[1.06;31.5]	**0.041**	
	nonplatinum	104 / 65	0.60	[0.30;1.20]	0.151	
**Menopausal status**						0.108
peri-post / pre	platinum	16 / 52	3.07	[0.62;23.4]	0.177	
	nonplatinum	92 / 77	0.69	[0.35;1.36]	0.287	
**BMI (kg/m^2)**						0.653
≥ 30 / < 30	platinum	9 / 59	4.05	[0.57;40.3]	0.166	
	nonplatinum	39 / 130	2.23	[0.99;5.17]	0.052	
**BRCA**						0.951
mutated / undetected	platinum	48 / 20	0.92	[0.12;4.79]	0.928	
	nonplatinum	24 / 145	1.04	[0.39;2.74]	0.941	
**Baseline cT**						**0.074**
T3-T4d / T1-T2	platinum	16 / 52	1.28	[0.31;5.98]	0.735	
	nonplatinum	44 / 125	0.27	[0.09;0.68]	**0.005**	
**Baseline cN**						0.567
N1-N3 / N0	platinum	33 / 35	0.80	[0.24;2.80]	0.723	
	nonplatinum	96 / 73	1.19	[0.60;2.38]	0.611	
**Grade**						0.585
3 / 2	platinum	57 / 8	0.99	[0.14;6.01]	0.989	
	nonplatinum	138 / 28	1.74	[0.69;4.68]	0.242	
**Ki-67**						**0.035**
≥ 65% / < 65%	platinum	48 / 18	5.15	[1.24;24.7]	0.024	
	nonplatinum	115 / 49	0.90	[0.42;1.94]	0.790	
**Dose-dense AC**						0.945
yes / no	platinum	19 / 49	0.84	[0.22;3.20]	0.791	
	nonplatinum	16 / 153	0.90	[0.28;2.77]	0.879	
**Early change from baseline cT**						0.171
undecreased from any T /	platinum	29 / 27	0.69	[0.13;4.10]	0.688	
decreased from T1-T2	nonplatinum	52 / 85	0.45	[0.15;1.26]	**0.010**	
decreased from T3-T4d /	platinum	12 / 27	1.57	[0.30;10.6]		
decreased from T1-T2	nonplatinum	32 / 85	0.24	[0.08;0.64]		
**Early change from baseline cN**						0.140
decreased from N1-N3 /	platinum	12 / 35	0.60	[0.14;2.82]	0.763	
N0	nonplatinum	53 / 73	2.00	[0.94;4.32]	**0.003**	
undecreased from N1-N3 /	platinum	21 / 35	1.17	[0.23;8.78]		
N0	nonplatinum	43 / 73	0.30	[0.08;0.91]		

Table S2. Univariable and multivariable analysis of pCR predictors according to NACT type for early responders. NACT, neoadjuvant chemotherapy; BMI, Body mass index

			Univariable analysis				Multivariable analysis			
	**NACT**	**N**	**OR**	**95% CI**	***p***	***p* for inter.**	**OR**	**95% CI**	***p***	***p* for inter.**
**NAT** platinum / nonplatinum		43 / 126	3.20	[1.52;7.16]						
**Age (10-years)**						0.187				
	platinum	43	1.29	[0.64;2.81]	0.480					
	nonplatinum	126	0.78	[0.58;1.02]	0.071					
**Age (years)**						**0.014**				**0.013**
≥ 45 / < 45	platinum	13 / 30	6.00	[0.96;117]	0.056		16.9	[1.54;721]	**0.017**	
	nonplatinum	75 / 51	0.53	[0.26;1.09]	0.086		0.75	[0.33;1.68]	0.481	
**Menopausal status**						0.222				
peri-post / pre	platinum	7 / 36	2.31	[0.33;46.5]	0.431					
	nonplatinum	67 / 59	0.61	[0.30;1.22]	0.162					
**BMI (kg/m^2)**						0.683				
≥ 30 / < 30	platinum	4 / 39	1.03	[0.12;22.2]	0.978					
	nonplatinum	29 / 97	1.78	[0.77;4.20]	0.176					
**BRCA**						0.911				
mutated / undetected	platinum	39 / 4	0.97	[0.04;8.59]	0.978					
	nonplatinum	18 / 108	1.12	[0.41;3.07]	0.827					
**Baseline cT**						0.082				
T3-T4d / T1-T2	platinum	9 / 34	1.26	[0.25;9.51]	0.793					
	nonplatinum	24 / 102	0.22	[0.07;0.61]	**0.003**					
**Baseline cN**						0.602				
N1-N3 / N0	platinum	17 / 26	0.72	[0.18;2.98]	0.643					
	nonplatinum	70 / 56	1.09	[0.54;2.21]	0.811					
**Grade**						**0.422**				
3 / 2	platinum	35 / 5	0.62	[0.03;4.91]	0.680					
	nonplatinum	103 / 21	1.66	[0.64;4.50]	0.298					
**Ki-67**						**0.014**				**0.004**
≥ 65% / < 65%	platinum	34 / 8	7.78	[1.51;47.6]	**0.014**		24.8	[2.59;770]	**0.004**	
	nonplatinum	89 / 34	0.79	[0.36;1.75]	0.568		0.77	[0.31;1.89]	0.574	
**Dose-dense AC**						0.790				
yes / no	platinum	13 / 30	1.21	[0.28;6.42]	0.803					
	nonplatinum	13 / 113	0.94	[0.29;2.99]	0.911					
**Early change from baseline cT**						0.211				0.841
undecreased from any T / decreased from T1-T2	platinum	7 / 27	0.88	[0.15;7.07]	0.957		0.26	[0.01;5.13]	0.558	
	nonplatinum	17 / 85	0.54	[0.18;1.54]	**0.006**		0.31	[0.09;1.06]	**0.011**	
decreased from T3-T4d / decreased from T1-T2	platinum	9 / 27	1.23	[0.23;9.53]			0.45	[0.04;6.63]		
	nonplatinum	24 / 85	0.20	[0.06;0.56]			0.21	[0.06;0.66]		
**Early change from baseline cN**						0.064				**0.020**
decreased from N1-N3 / N0	platinum	12 / 26	0.60	[0.13;2.87]	0.767		0.57	[0.04;7.57]	0.266	
	nonplatinum	52 / 56	1.85	[0.86;4.02]	**<0.001**		2.73	[1.14;6.93]	**0.003**	
undecreased from N1-N3 / N0	platinum	5 / 26	1.20	[0.14;25.9]			6.96	[0.39;407]		
	nonplatinum	18 / 56	0.14	[0.02;0.57]			0.27	[0.04;1.23]		
